# Long-acting recombinant human follicle-stimulating hormone (SAFA-FSH) enhances spermatogenesis

**DOI:** 10.3389/fendo.2023.1132172

**Published:** 2023-02-23

**Authors:** Daham Kim, Soohyun Lee, Yoon Hee Cho, Min Jeong Kang, Cheol Ryong Ku, Hyunjin Chi, Jungsuk Ahn, Kyungsun Lee, Jaekyu Han, Susan Chi, Moo Young Song, Sang-Hoon Cha, Eun Jig Lee

**Affiliations:** ^1^ Department of Internal Medicine, Institute of Endocrine Research, Yonsei University College of Medicine, Seoul, Republic of Korea; ^2^ AprilBio Co., Ltd., Rm 602, Biomedical Science Building, Kangwon National University, Chuncheon, Republic of Korea

**Keywords:** FSH, gonadotrophin replacement therapy, hypogonadotropic hypogonadism, infertility, testis

## Abstract

**Introduction:**

Administration of follicle-stimulating hormone (FSH) has been recommended to stimulate spermatogenesis in infertile men with hypogonadotropic hypogonadism, whose sperm counts do not respond to human chorionic gonadotropin alone. However, FSH has a short serum half-life requiring frequent administration to maintain its therapeutic efficacy. To improve its pharmacokinetic properties, we developed a unique albumin-binder technology, termed “anti-serum albumin Fab-associated” (SAFA) technology. We tested the feasibility of applying SAFA technology to create long-acting FSH as a therapeutic candidate for patients with hypogonadotropic hypogonadism.

**Methods:**

SAFA-FSH was produced using a Chinese hamster ovary expression system. To confirm the biological function, the production of cyclic AMP and phosphorylation of ERK and CREB were measured in TM4-FSHR cells. The effect of gonadotropin-releasing hormone agonists on spermatogenesis in a hypogonadal rat model was investigated.

**Results:**

In in vitro experiments, SAFA-FSH treatment increased the production of cyclic AMP and increased the phosphorylation of ERK and CREB in a dose-dependent manner. In animal experiments, sperm production was not restored by human chorionic gonadotropin treatment alone, but was restored after additional recombinant FSH treatment thrice per week or once every 5 days. Sperm production was restored even after additional SAFA-FSH treatment at intervals of once every 5 or 10 days.

**Discussion:**

Long-acting FSH with bioactivity was successfully created using SAFA technology. These data support further development of SAFA-FSH in a clinical setting, potentially representing an important advancement in the treatment of patients with hypogonadotropic hypogonadism.

## 1 Introduction

Hypogonadotropic hypogonadism (HH) is associated with decreased secretion of the gonadotropins, luteinizing hormone and follicle-stimulating hormone (FSH), resulting in deficiency in both testosterone and spermatogenesis (1). A logical approach for fertility induction in men with HH is to replace gonadotropin-releasing hormone (GnRH) in a pulsatile manner (2). However, this approach is not effective in patients with primary pituitary disease and is limited by the availability of drugs and suitable infusion devices capable of delivering pulsatile GnRH (3). Importantly, fertility outcomes are similar between pulsatile GnRH and gonadotrophin replacement therapies (4). Therefore, gonadotrophin replacement therapy is the most common approach for fertility induction in men with HH.

In practice, monotherapy with human chorionic gonadotropin (hCG) (2000 IU thrice per week), which has the biological activity of luteinizing hormone but a longer half-life in circulation, may be sufficient for the stimulation of spermatogenesis. However, hCG monotherapy is less successful in men who lack testicular development (5). If sperm counts do not respond to hCG alone, FSH (75–300 IU thrice per week) should be added to the regimen in the form of highly purified urinary gonadotropins or recombinant FSH (2, 6). FSH pretreatment followed by combination treatment with hCG and FSH appears to improve fertility in a subset of men with severe HH (7).

Owing to its short half-life, FSH needs to be injected multiple times per week for extended periods, from several months to years. The inconvenience of repeated injections is a burden to patients and hence treatment with fewer injections may be more favorable and result in fewer medication errors and improved adherence (8). Therefore, over the past decades, several different approaches have been used to generate FSH preparations with different pharmacokinetic profiles, particularly to reduce the frequency of administration (9, 10). Corifollitropin alfa, the longest-acting FSH available in clinics, has an approximately 1.5- to 2-fold longer elimination half-life than FSH (9). Previous studies have shown that long-acting FSH can effectively and safely replace FSH in the treatment regimen of adult men with HH desiring improved fertility (8, 11).

We previously developed a unique albumin-binder technology that profoundly extended the half-life of a therapeutic protein, termed “anti-serum albumin Fab-associated” (SAFA) technology (12, 13). Using this technology, a long-acting recombinant human interferon beta (at least 2-fold longer serum half-life in rats and monkeys) for multiple sclerosis and a long-acting feline granulocyte colony-stimulating factor (approximately 5-fold longer serum half-life in cats) were developed (14, 15). In the present study, we tested the feasibility of applying SAFA technology to develop long-acting FSH as a therapeutic candidate for patients with HH.

## 2 Materials and methods

### 2.1 SAFA-FSH production and purification

SAFA-FSH was produced by CHO glutamine synthetase null^−/−^ K1 cell (Horizon Discovery, Waterbeach, UK) and purified using a three step purification protocol—capture for affinity chromatography, intermediate purification for multimodal chromatography, and polishing for cation exchange chromatography, as described previously (14, 15). After purification, sodium dodecyl sulfate-polyacrylamide gel electrophoresis (SDS-PAGE) and size-exclusion high-performance liquid chromatography (SE-HPLC) under native conditions were used to determine the apparent molecular weight and purity.

### 2.2 Cell culture

TM4 cells, a mouse Sertoli cell line, were purchased from the Korean Cell Line Bank (KLCB No. 21715; Seoul, South Korea). 293FT cells were purchased from Invitrogen (Carlsbad, CA, USA). Cells were cultured in Dulbecco’s Modified Eagle’s medium (Gibco, Thermo Fisher Scientific, Waltham, MA, USA) supplemented with 10% fetal bovine serum (Hyclone, Logan, UT, USA) and 1% penicillin/streptomycin (Hyclone) in a humidified atmosphere of 5% CO_2_ and 95% air at 37°C.

### 2.3 Plasmid construction and stable cell line generation

The human FSH receptor gene was inserted into the lentiviral vector pLECE3-Green Fluorescent Protein (GFP) using the HpaI/NotI restriction sites to generate pLECE3-hFSHR-GFP. Lentiviral particles were generated using three plasmids, VSVG, RSV-REV, and PMDLg/pPRE, in HEK293FT cells cotransfected with pLECE3-hFSHR-GFP. Cells were transfected using polyjet transfection reagent (SignaGen Laboratories, Frederick, MD, USA). Two days after transfection, the culture medium was harvested and sterilized using a 0.45 μm syringe filter. Purified lentiviral particles were used to infect TM4 cells. Three days after infection, cells were separated from GFP-positive single cells into a 96-well plate using BD LSRFortessa (Becton Dickinson, Franklin Lakes, NJ, USA).

### 2.4 Measurement of cyclic AMP (cAMP) production

TM4 and TM4-FSHR cells were seeded (0.3 × 10^6^ cells/plate) and cultured in 6 cm plates. The next day, after starvation for 4 h, the cells were stimulated with recombinant FSH (Follitrope, LG Chem, Seoul, South Korea) or SAFA-FSH at different concentrations in 0.5 mM isobutylmethylxanthine (Sigma-Aldrich, St. Louis, MO, USA) for 15 min at 37°C. The reaction was stopped by aspirating the medium and washing twice with cold Dulbecco’s phosphate-buffered saline (DPBS). Cells were lysed with 80 μL of lysis buffer and incubated on ice for 30 min. Cell lysates were obtained by centrifugation at 14000 ×*g* for 10 min at 4°C. cAMP concentrations were measured using a cAMP XP assay kit (Cell Signaling Technology, Danvers, MA, USA). In each experiment, a standard curve was generated and used to calculate the cAMP concentration.

### 2.5 Western blotting

Whole cell lysates were prepared, and the assay was carried out according to standard procedures. Cells were washed twice with cold DPBS and lysed in lysis buffer containing 1 mM phenylmethylsulfonyl fluoride and a 1× protease inhibitor cocktail (Sigma-Aldrich). The lysates were centrifuged at 12000 rpm for 15 min at 4°C, and the supernatants obtained were used for analysis. The protein concentration was determined using the Pierce BCA Protein Assay Kit (Thermo Fisher Scientific). Equal quantities of protein were loaded onto Bolt 4–12% Bis-Tris Plus Gels (Invitrogen) and separated at 200 V for 35 min. Proteins were transferred onto polyvinylidene fluoride membranes (Invitrogen) using a Power Blotter (Invitrogen). The membranes were blocked with EveryBlot blocking buffer (Bio-Rad, Hercules, CA, USA) for 15 min at 24°C. Membranes were incubated with the following primary antibodies for 1 h at 24°C: rabbit anti-CREB (1:1000), rabbit anti-phospho CREB (1:1000), mouse anti-ERK (1:2000), and rabbit anti-phospho ERK (1:2000). Primary antibodies were purchased from Cell Signaling Technology. Blots were washed six times for 5 min with Tris-buffered saline containing 0.05% Tween 20 and incubated with horseradish peroxidase (HRP)-conjugated anti-mouse IgG or anti-rabbit IgG secondary antibodies (1:2000, Thermo Fisher Scientific) for 1 h at 24°C. Immunoreactivity was detected with Amersham ECL (Cytiva, Marlborough, MA, USA) using an iBright 1500 (Invitrogen). The intensity of the protein bands was quantified using iBright analysis and normalized to β-actin in each sample.

### 2.6 Animal experimental design

Male Sprague-Dawley (SD) rats were purchased from Orient Bio (Seoul, South Korea). The animals were maintained under controlled conditions (22°C, 12 h light, 12 h dark cycle) and received rodent chow and tap water. The animals were acclimatized for 1 week prior to the study.

For the pharmacokinetic study, recombinant FSH (88 μg/kg) (Group 1), SAFA-FSH (200 μg/kg) (Group 2), and SAFA-FSH (600 μg/kg) (Group 3) were injected intravenously into 8-week-old male SD rats (n = 5 in each group). The dosing concentration for Group 2 (SAFA-FSH, 200 μg/kg) was determined to adjust the approximate equivalent molar ratio compared to recombinant FSH, whereas that for Group 3 (SAFA-FSH, 600 μg/kg) was determined to confirm the dose-dependent behavior of SAFA-FSH. Blood samples were collected at a predetermined time, and the plasma concentrations of recombinant FSH and SAFA-FSH were determined by quantitative enzyme-linked immunosorbent assay (ELISA). Briefly, an ELISA plate (Greiner Bio-One, Kremsmünster, Austria) was coated with a mouse anti-human FSH beta Antibody (100 ng/well) for 16 h at 4°C using a carbonated coating buffer (pH 9.6), followed by treating with a Starting Block buffer (Thermo Fisher Scientific) at 25°C for 3 h. After washing the plates, the appropriately diluted serum samples and standards were aliquoted into each well of the plate, followed by incubation at 25°C for 2 h. After washing the plates, a mouse anti-human FSH alpha-biotinylated Antibody (Bio-Rad) was aliquoted and incubated at 25°C for 1 h. After washing again, a Pierce™ high sensitivity streptavidin-HRP was added to each well, and the TMB substrate (Surmodics, Eden Prairie, MN, USA) was added to react with HRP. Finally, 1 N hydrochloric acid (Daejung Chemicals, Siheung, South Korea) was added into each well to quench the reaction and the absorbance at 450 nm was measured using a microplate reader (BMG LABTECH, Ortenberg, Germany). The pharmacokinetic parameters were evaluated Phoenix^®^ WinNonlin^®^ (Ver. 8.1, Certara, Princeton, NJ, USA). Meanwhile, the pharmacokinetic study was performed by KNOTUS Co., Ltd. (Incheon, South Korea).

**Figure 1:**
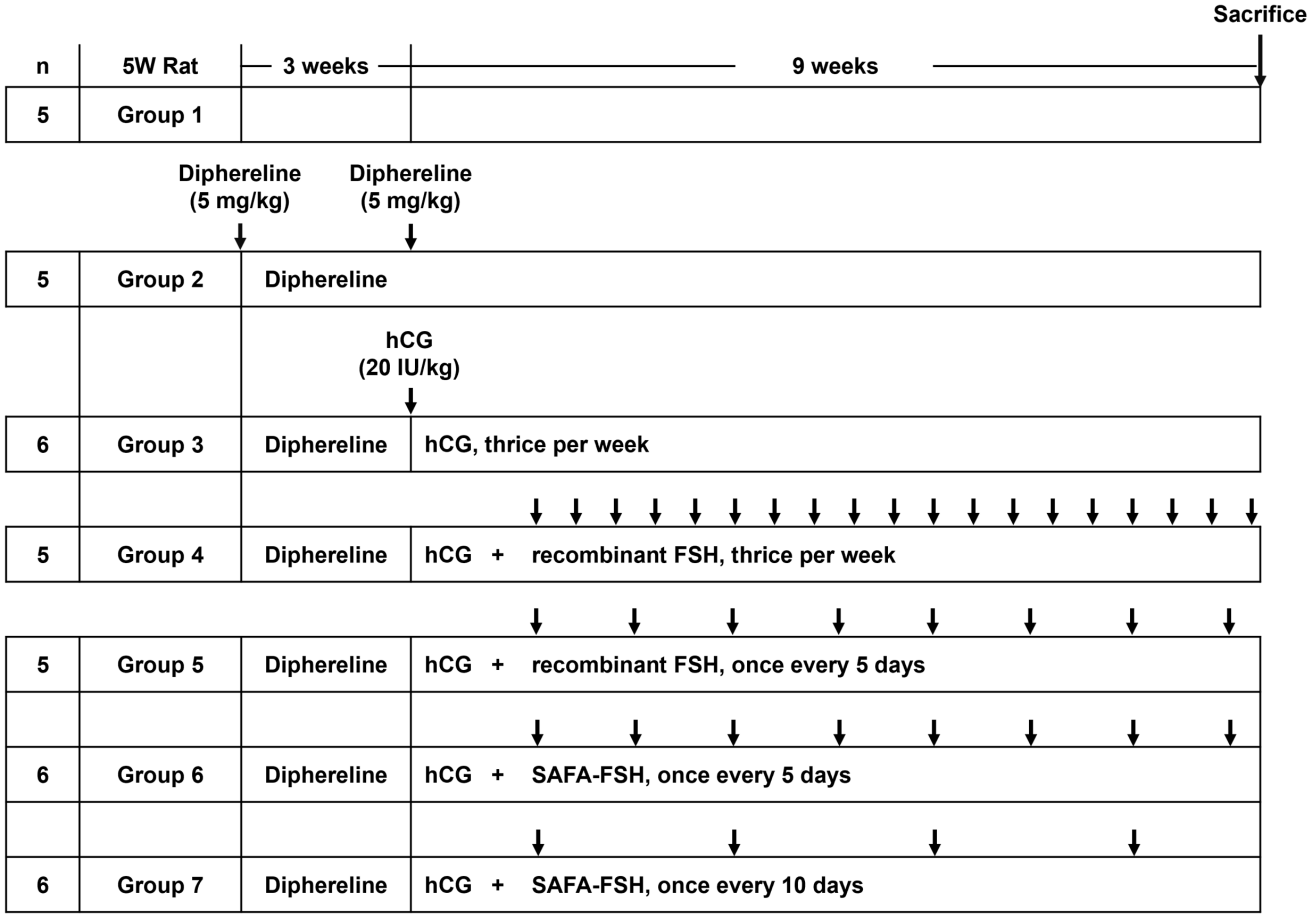
Time course of Sprague-Dawley (SD) rat *in vivo* experiments per group. Arrows indicate the injection cycle of each drug. FSH, follicle-stimulating hormone; hCG, human chorionic gonadotropin; SAFA, anti-serum albumin Fab-associated.

For the pharmacodynamic study, 5-week-old male SD rats were randomly divided into seven groups (n = 5–6 in each group) based on body weight and two rats were housed per cage. Body weight was measured twice per week to record weight gain. Rats received subcutaneous injections of Diphereline (5 mg/kg, Ipsen, Paris, France) at the start of the experiment and 3 weeks later to ensure suppressed production of gonadotropin in all groups, except for Group 1 (control) (**Figure 1**). Subsequently, hCG (20 IU/kg, LG Chem) was injected subcutaneously thrice per week in all groups, except for Groups 1 and 2 (Diphereline only) for approximately 9 weeks. Groups 1 and 2 were injected with saline instead of drugs. Additional recombinant FSH (25 IU/kg = 1.85 μg/kg) was injected subcutaneously thrice per week (Group 4) or once every 5 days (Group 5). Additional SAFA-FSH (9.87 μg/kg, thrice more than recombinant FSH when considering the molecular weight) was injected subcutaneously once every 5 days (Group 6) or once every 10 days (Group 7). The rats were sacrificed after 9 weeks of hCG and FSH injections. The Institutional Animal Care and Use Committee (IACUC, Yonsei University Health System) approved the study protocol (approval number 2021-0087).

### 2.7 Testosterone measurement

Serum testosterone levels were measured weekly from 1 week after the first Diphereline injection. Blood samples were collected from the orbital sinus using capillary tubes after anesthesia at the same time every week, and only serum was isolated by centrifugation at 3000 rpm for 10 min at 4°C. Testosterone levels were measured by Seoul Clinical Laboratories (SCL, Yongin, South Korea) using an electrochemiluminescence assay (ECLIA, Roche Diagnostics, Indianapolis, IN, USA).

### 2.8 Sperm count

Sperm was collected from the epididymis cauda. The cauda was placed in a 6 cm plate with 10 mL DPBS and minced with a scalpel, allowing sperm to be dispersed in saline and incubated for 15 min at 37°C under 5% CO_2_. The mixture was heated at 60°C for 1 min and then cooled to 24°C. The dispersed sperm solution was diluted appropriately to increase the accuracy of the sperm count. Sperms were counted and evaluated using a Neubauer hemocytometer (INCYTO, Cheonan, South Korea).

### 2.9 Statistical analysis

IBM SPSS Statistics version 25 (IBM, Armonk, NY, USA) was used for all statistical analyses. Data are presented as mean ± standard error of the mean. Statistical significance was determined using the Mann–Whitney U test. Differences were considered statistically significant at P < 0.05.

## 3 Results

### 3.1 SAFA-FSH characterization

**Figure 2:**
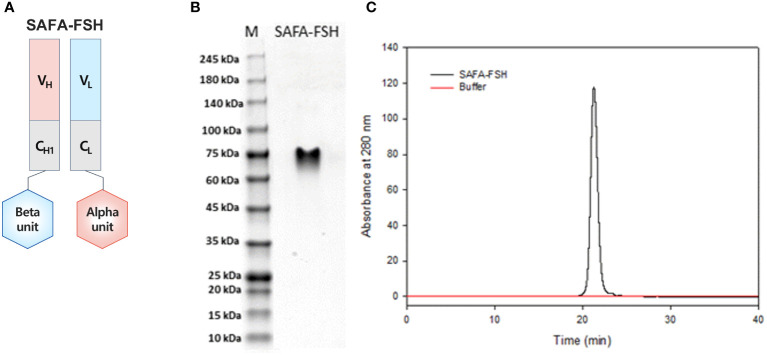
Anti-serum albumin Fab-associated (SAFA)-follicle-stimulating hormone (FSH) characterization. **(A)** Illustration of SAFA-FSH. **(B)** Sodium dodecyl sulfate–polyacrylamide gel electrophoresis under the non-reducing condition. The protein bands were visualized by using Coomassie Blue staining. **(C)** Size-exclusion high-performance liquid chromatography under the native condition. Elution was monitored using UV absorption at 280 nm. V, variable region; C, constant region; H, heavy chain; L, light chain.

SAFA-FSH, a long-acting recombinant human FSH, is shown in **Figure 2A**. SAFA-FSH is a protein composed of two subunits, alpha and beta subunits, which are bound to the light and heavy chain in the Fab, respectively. Using SDS–PAGE under non-reducing conditions, SAFA-FSH appeared as a single band by Coomassie Blue staining, with a molecular weight between 60 and 75 kDa (**Figure 2B**). The average molecular mass of SAFA-FSH was approximately 1.78-fold greater than that of recombinant FSH (non-reduced 71 versus 40 kDa) as the molecular weight of the SAFA region is approximately 50 kDa. The purity of the SAFA-FSH used in our study was > 95%, as determined by SE-HPLC (**Figure 2C**).

### 3.2 *In vitro* functional assays

**Figure 3:**
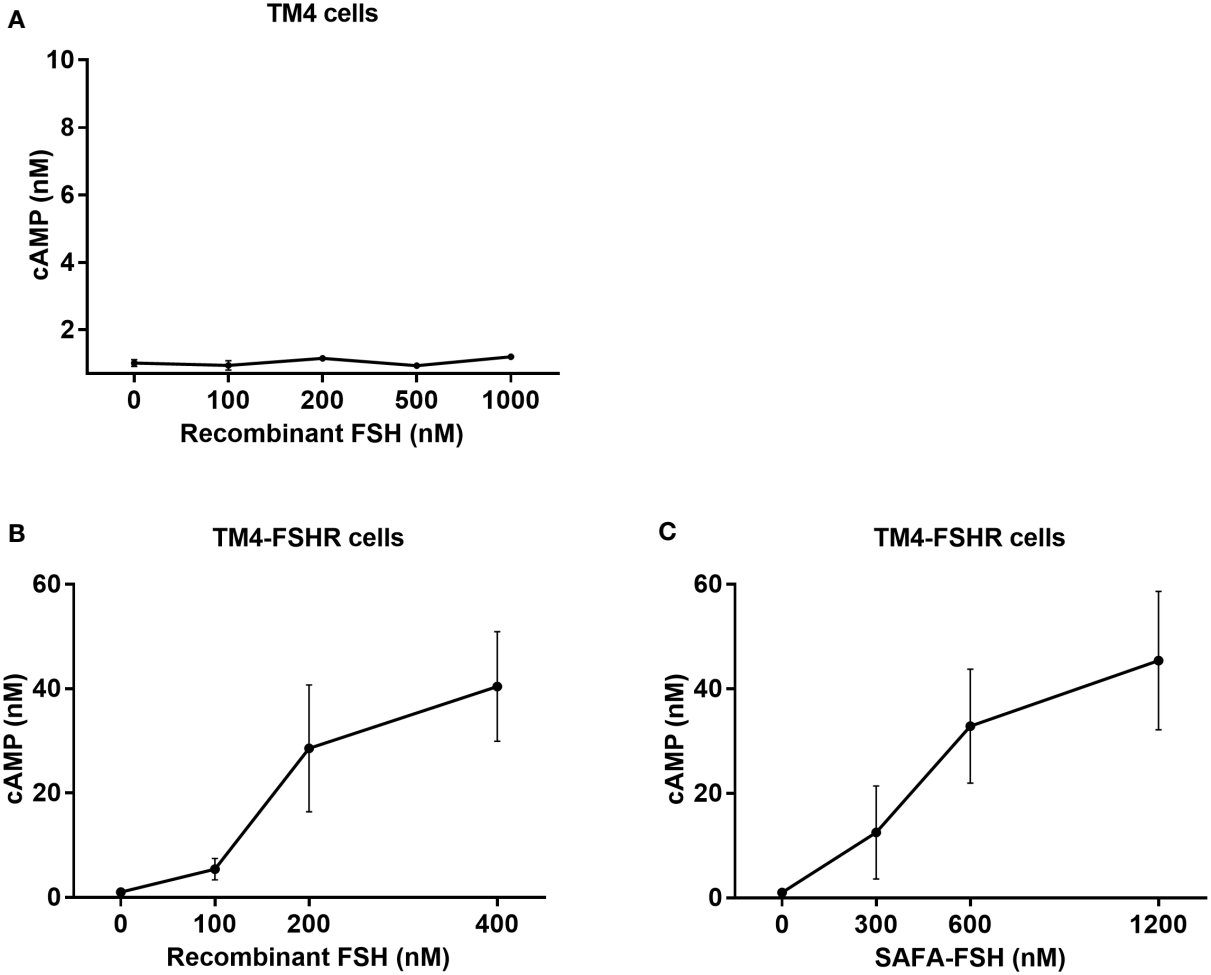
Cyclic adenosine monophosphate (cAMP) production for recombinant follicle-stimulating hormone (FSH) or anti-serum albumin Fab-associated (SAFA)-FSH *in vitro*. **(A)** cAMP production of TM4 cells treated with recombinant FSH at indicated dose. **(B, C)** cAMP production of TM4-FSHR cells treated with recombinant FSH or SAFA-FSH at indicated doses. Line plots indicate the mean ± SEM of three independent experiments.

To assess the bioactivity of SAFA-FSH versus recombinant FSH *in vitro*, TM4 cells were treated with different doses of recombinant FSH, and intracellular cAMP levels were measured by ELISA. However, the concentration of cAMP did not increase, regardless of recombinant FSH concentration (**Figure 3A**). Additional FSH treatment experiments could not be performed in TM4 Cells. Therefore, TM4-FSHR cells were generated by overexpression of the *FSHR* gene using lentivirus in TM4 cells and were treated with recombinant FSH or SAFA-FSH at different concentrations. Unlike in TM4 cells, as the concentration of FSH increased, cAMP concentration increased in a dose-dependent manner (**Figures 3B, C**). The effect of SAFA-FSH was lower than that of recombinant FSH when treated with similar concentrations of FSH. Calculating the dose needed to achieve a similar cAMP level indicated that thrice more SAFA-FSH than recombinant FSH was required, demonstrating a considerable difference in the relative bioactivity of the two compounds.

**Figure 4:**
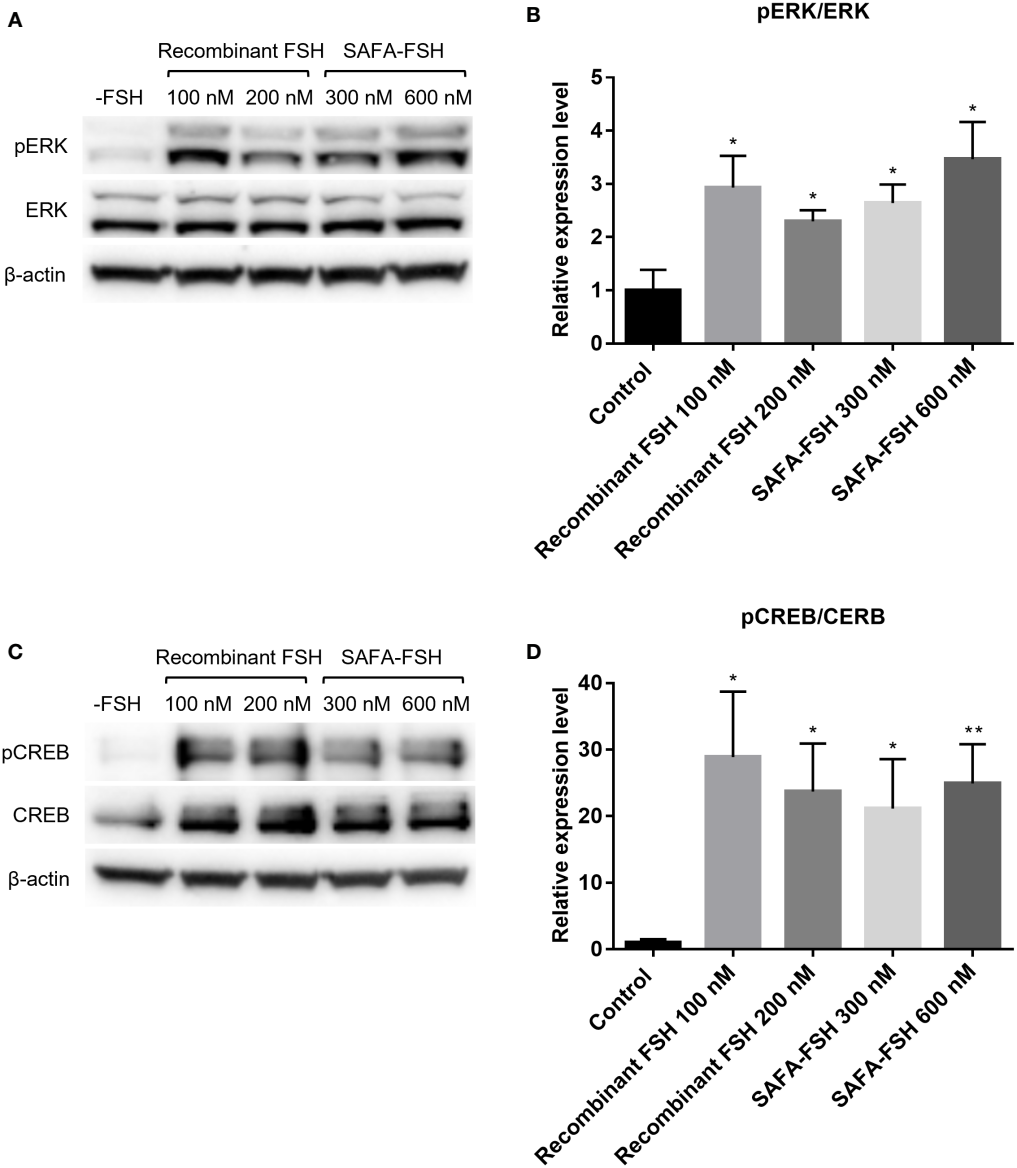
Protein expression for recombinant follicle-stimulating hormone (FSH) or anti-serum albumin Fab-associated (SAFA)-FSH *in vitro*. **(A)** Representative western blot analysis of protein extracts from TM4-FSHR cells showing the levels of ERK and pERK. **(B)** Relative ERK activity was derived as pERK normalized to ERK. **(C)** Representative western blot analysis of protein extracts from TM4-FSHR cell showing the levels of CREB and pCREB. **(D)** Relative CREB activity was derived as pCREB normalized to CREB. Each sample was normalized with β-actin. Data are representative of four independent experiments and are presented as mean ± standard error of mean (SEM). *P < 0.05, **P < 0.01 versus control.

Moreover, the molecular responses stimulated by recombinant FSH or SAFA-FSH in TM4-FSHR cells were analyzed by detecting cAMP-related signaling molecules by assessing ERK and CREB activation, i.e., phosphorylation. When TM4-FSHR cells were treated with recombinant FSH or SAFA-FSH at different concentrations of two points, the phosphorylation level of ERK was higher than that of the negative control (**Figures 4A, B**). Likewise, when TM4-FSHR cells were treated with recombinant FSH or SAFA-FSH, CREB phosphorylation increased expectedly (**Figures 4C, D**). Both recombinant FSH and SAFA-FSH increased phosphorylation compared to the control; however, the increase noted with recombinant FSH was not concentration dependent. These results suggest that SAFA-FSH has the same effect on the signaling pathway as recombinant FSH.

### 3.3 *In vivo* pharmacokinetic assays

**Figure 5:**
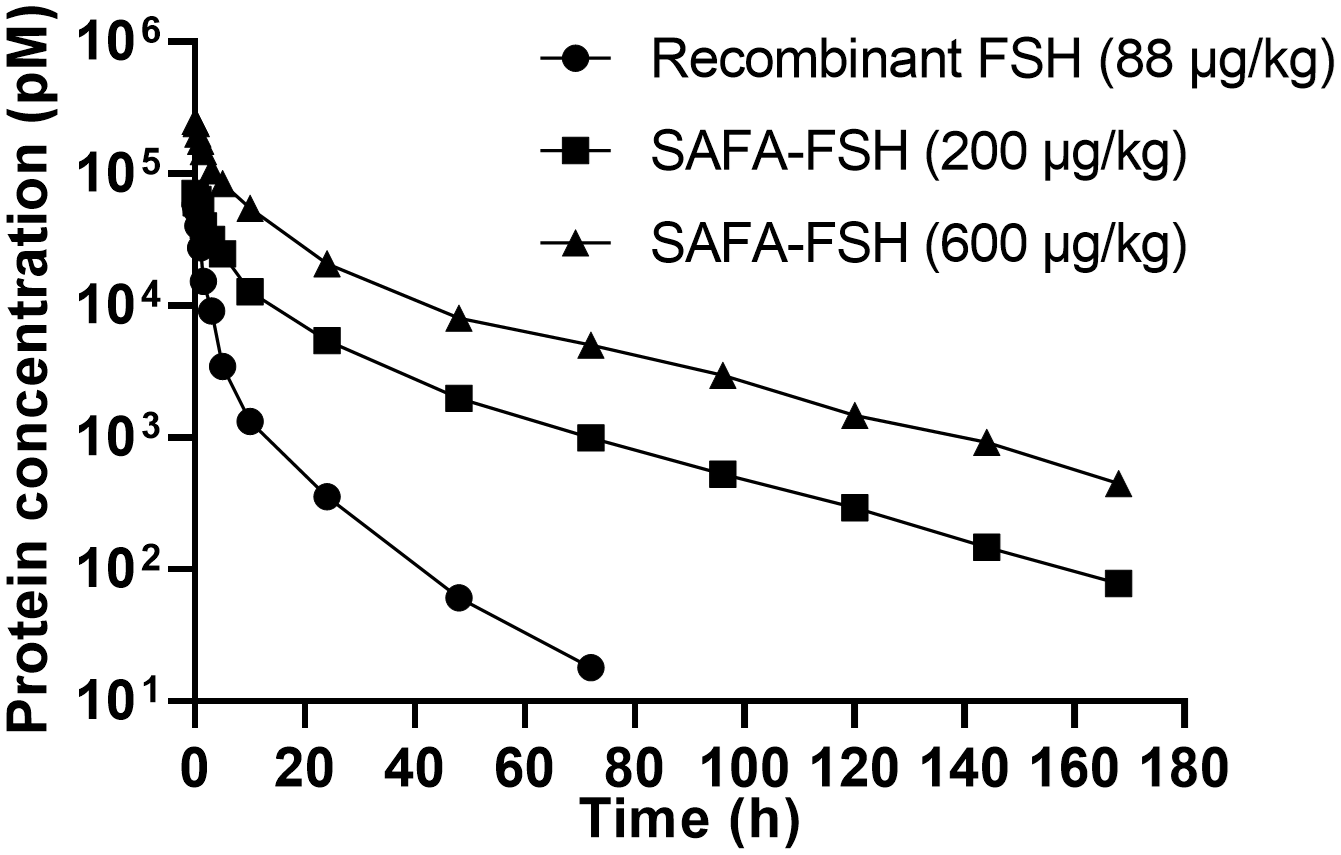
Pharmacokinetics of anti-serum albumin Fab-associated (SAFA)-follicle-stimulating hormone (FSH) in rats.

The pharmacokinetic profiles of SAFA-FSH were studied using a rat model that was administered a single intravenous injection. Recombinant FSH was used in this study as a reference molecule. As shown in **Figure 5**, the high concentration of SAFA-FSH was maintained for much longer than that of recombinant FSH, and the pharmacokinetic parameters revealed that SAFA-FSH had an approximately 2.7-fold longer serum half-life than recombinant FSH (t_1/2_ = 27.1–29.3 h vs. 10.4 h) (**Table 1**). In the case of T_max_ values, SAFA-FSH showed T_max_ = 0.08–0.12 h and recombinant FSH showed T_max_ = 0.08 h, suggesting that SAFA-FSH enters the blood circulation similar to recombinant FSH. C_max_ values were 68292.8–252519.4 pM for SAFA-FSH and 57431.6 pM for recombinant FSH, and renal clearance (CL) rates were 0.00028–0.00041 μg/(h × pM)/kg for SAFA-FSH and 0.00078 μg/(h × pM)/kg for recombinant FSH. In the case of AUC_inf_, SAFA-FSH showed 4.5- to 19-fold higher value than recombinant FSH (505688.6–2142357.8 h × pM vs. 113565.0 h × pM), indicating the sustained bioavailability of SAFA-FSH.

**Table 1:** Pharmacokinetic parameters for subcutaneous administration of recombinant FSH and SAFA-FSH in SD rats.

Parameters	Recombinant FSH (88 µg/kg)	SAFA-FSH (200 µg/kg)	SAFA-FSH (600 µg/kg)
C_max_ (pM)	57431.6	68292.8	252519.4
T_max_ (h)	0.08	0.08	0.12
AUC_last_ (h × pM)	113291.1	502616.4	2123006.7
AUC_inf_ (h × pM)	113565.0	505688.6	2142357.8
T_1/2_ (h)	10.4	27.1	29.3
CL (μg/(h × pM)/kg)	0.00078	0.00041	0.00028
AUC_ext_ (%)	0.2	0.6	0.9

SD, Sprague-Dawley; FSH, follicle-stimulating hormone; SAFA, anti-serum albumin Fab-associated; C_max_, maximum concentration; T_max_, time to reach C_max_; AUC_last_, area under the curve to last measurable concentration; AUC_inf_, area under the curve to time infinity; T_1/2_, biological half-life; CL, clearance rate; AUC_ext_, extrapolated area under the curve, [(AUC_inf_ – AUC_last_)/AUC_inf_] × 100.

### 3.4 *In vivo* functional assays

**Figure 6:**
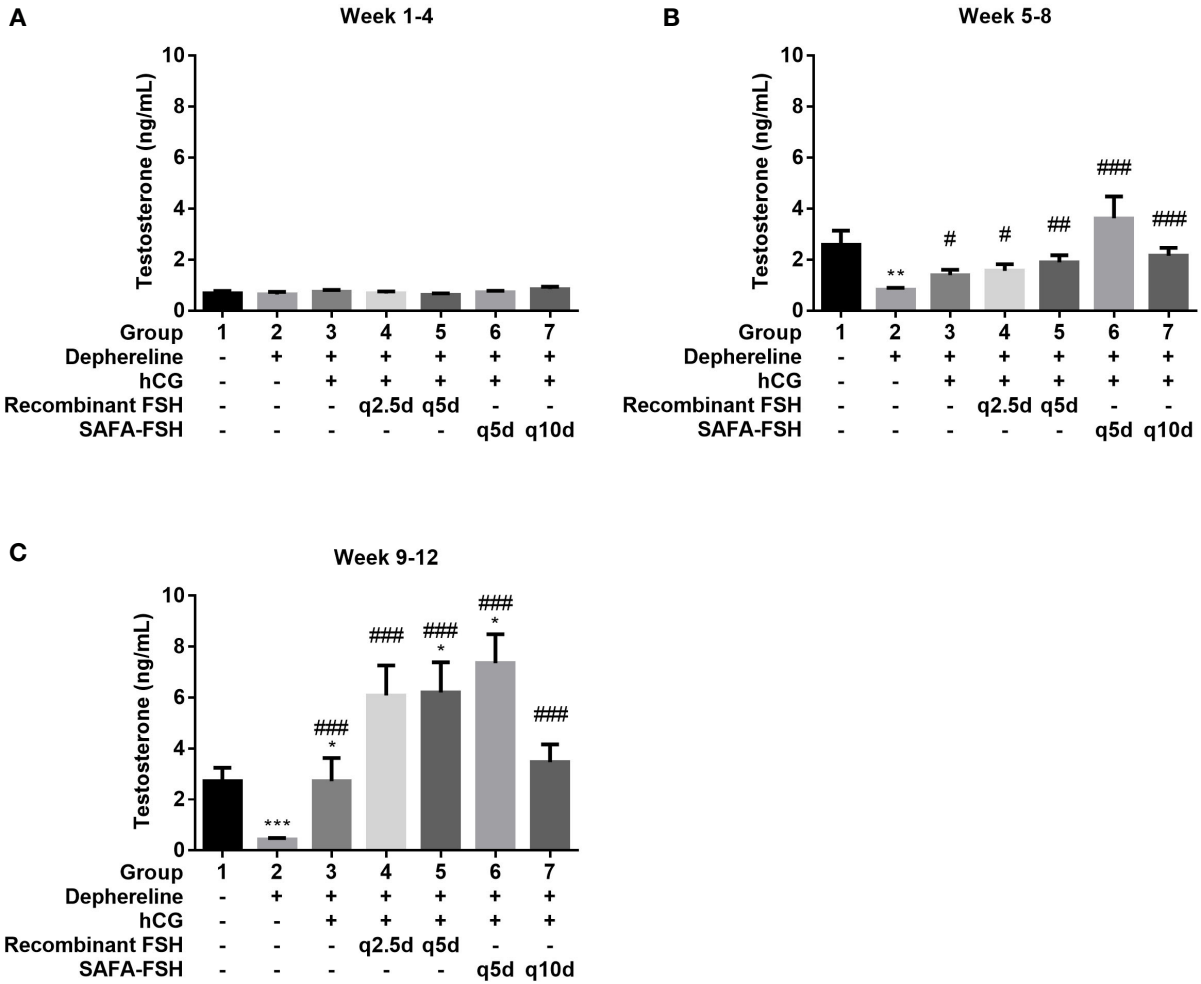
Mean serum testosterone level. **(A)** Week 1–4, **(B)** Week 5–8, and **(C)** Week 9–12. Data are presented as mean ± SEM. *P < 0.05, **P < 0.01, ***P < 0.001 versus Group 1. ^#^P < 0.05, ^##^P < 0.01, and ^###^P < 0.001 versus Group 2. FSH, follicle-stimulating hormone; hCG, human chorionic gonadotropin; SAFA, anti-serum albumin Fab-associated.

To confirm the function of SAFA-FSH as a long-acting hormone, the GnRH agonist Diphereline was injected into male rats to suppress the production of gonadotropin and create an animal model of hypogonadism. Rats were then treated with recombinant FSH or SAFA-FSH. There was no difference in body weight between the groups during the study period. There was no difference in mean serum testosterone levels in all groups during weeks 1–4 because the rats were adolescent (**Figure 6A**). At weeks 5–8, the mean serum testosterone level was elevated in Group 1 (**Figure 6B**). However, the mean serum testosterone level in Group 2 was lower than that in Group 1, implying that the hypogonadism model was well established in male SD rats. Compared with Group 2, Groups 3–7, which received hCG, showed an increase in the mean serum testosterone level. In weeks 9–12, the mean serum testosterone level in Group 2 remained low when compared with that in Group 1. This suggests that the hypogonadism animal model continued to be well-maintained. Compared to Group 2, Groups 3–7 still showed an increase in mean serum testosterone levels. The mean serum testosterone levels in Groups 5 and 6 were higher than those in Group 1. In weeks 9–12, the mean serum testosterone level in Group 2 remained low when compared with that in Group 1 (**Figure 6C**).

**Figure 7:**
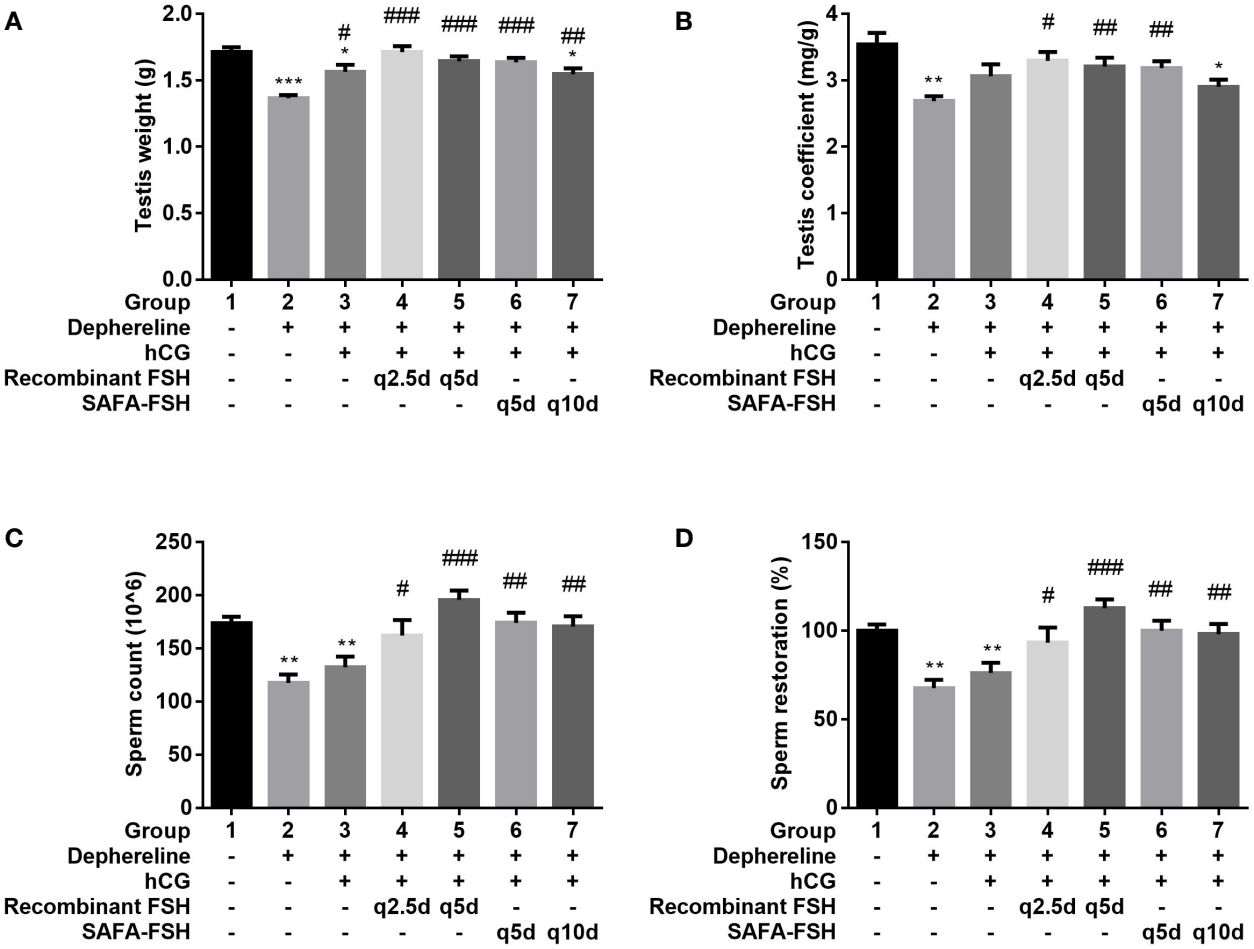
Effect of recombinant follicle-stimulating hormone (FSH) or anti-serum albumin Fab-associated (SAFA)-FSH on spermatogenesis in hypogonadism rats. **(A)** Testis weight, **(B)** Testis coefficient, **(C)** Sperm count, and **(D)** Sperm restoration. Data are presented as mean ± SEM. *P < 0.05, **P < 0.01, ***P < 0.001 versus Group 1. ^#^P < 0.05, ^##^P < 0.01, and ^###^P < 0.001 versus Group 2. hCG, human chorionic gonadotropin.

After 9 weeks of hCG and FSH injection, the rats were sacrificed, and testis were weighed. The testis weights were lower in Groups 2, 3, and 7 than in Group 1 (**Figure 7A**) and were higher in Groups 3–7 than in Group 2. The testis coefficient (testis/body weight ratio) values also showed similar results (**Figure 7B**). The total number of sperms in Group 2 was reduced by 68% compared with that in Group 1 (**Figures 7C, D**). Group 3 did not show restoration compared to Group 2. However, sperm production was restored after additional recombinant FSH treatment at intervals of thrice per week (Group 4) or once every 5 days (Group 5). Sperm production was restored even after additional SAFA-FSH treatment at intervals of once every 5 days (Group 6) or once every 10 days (Group 7). These results suggest that even when SAFA-FSH was injected over a longer cycle than recombinant FSH, sperm production was restored in a rat model of hypogonadism.

## 4 Discussion

Peptide hormones typically have short circulatory half-lives due to their rapid clearance from circulation. At the clinical level, frequent peptide hormone injections are required, which cause considerable discomfort to the patient (16). Thus, there is a need for technologies that can prolong the half-life of peptide hormones while maintaining high pharmacological efficacy. Long-acting growth hormones, a leader in the development of long-acting hormones, have been or are currently being developed in various ways to improve compliance (17). According to GlobalData’s recent report (Report Code: GDHCOA002), long-acting growth hormones will account for over 90% of the growth hormone deficiency market share across the US, Germany, and Japan by 2030. However, for peptide hormones other than growth hormones, continuous developmental research is not being actively conducted.

Corifollitropin alfa, a long-acting FSH that is currently available clinically, is indicated for controlled ovarian stimulation in combination with a GnRH antagonist for the development of multiple follicles in women undergoing fertility treatment and for treatment in adolescent men with HH in combination with hCG (18). Although corifollitropin alfa has a long half-life, 7 days after its injection, daily recombinant FSH injections should be continued until the criterion for triggering final oocyte maturation in women has been met. Its half-life is not long enough to sustain the entire therapeutic period. Corifollitropin alfa should be administered once every 2 weeks in combination with hCG injections twice weekly in men with HH (11). We attempted to apply SAFA technology to invent long-acting biologics using FSH as a model. It is expected to have a half-life of approximately 2 weeks in humans, and if development is successful, it is expected that additional daily recombinant FSH administration will not be required in women and longer dosing intervals are expected in men. SAFA can cross-react with serum albumin from various species, including cynomolgus monkeys and rats, implying that SAFA or its derivatives can be assessed using animal models in a preclinical setting (12).

In the present study, we generated SAFA-FSH, a long-acting recombinant human FSH. We also investigated the bioactivity of SAFA-FSH, both *in vitro* and *in vivo*. To confirm the applicability of SAFA-FSH, we investigated *in vitro* cell activation using recombinant FSH or SAFA-FSH in FSH receptor-expressing cells; the FSH receptor is expressed in TM4 cells (19). As the FSH receptor is a G protein-coupled receptor, it was expected that cAMP would increase when FSH was administered (20). cAMP-related signaling involves ERK and CREB activation (21). In Sertoli cells, cAMP binds to protein kinase A and mediates ERK phosphorylation (22). However, TM4 cells did not respond well to recombinant FSH stimulation. TM4 cells are reported to respond to FSH with an increase in cAMP production, but the FSH responsiveness is much reduced compared to primary Sertoli cell cultures (23). Unlike freshly prepared Sertoli cells, which expressed abundant FSH receptors, both primary cultured Sertoli cells and Sertoli cell lines express very low levels of FSHR (24). Likewise, when the expression level of FSHR in TM4 cells was confirmed by quantitative PCR, the expression level of FSHR was very low. Therefore, we created TM4-FSHR cells by overexpressing the *FSHR* gene and confirmed that cAMP levels and phosphorylation of ERK and CREB were increased by treatment with recombinant FSH or SAFA-FSH, respectively. These results clearly indicated that recombinant FSH and SAFA-FSH had the same effect on the signaling pathway. The bioactivity of SAFA-FSH seemed to be approximately 3-fold lower than that of recombinant FSH when considering the molecular weight, implying that the fusion of FSH to SAFA might slightly interfere with the biological activity of FSH, probably due to steric hindrance.

In our pharmacokinetic experiments using a rat model, the t_1/2_ of SAFA-FSH in rats after a single dose was approximately 2.7-fold greater at 27.1–29.3 h. SAFA-FSH displays a prolonged kinetic profile due to the presence of human anti-serum albumin Fab by utilizing the neonatal Fc receptor recycling mechanism similar to other albumin binders (12, 25, 26). However, since SAFA technology combines the Fab antibody fragment that binds to human serum albumin with high affinity, the half-life in rats is inevitably lower than that in humans. Of course, its half-life is longer than that of recombinant FSH, but direct comparison with humans is likely to be difficult. Considering that the serum half-life of endogenous rat serum albumin is approximately 46 h and a previous unpublished pharmacokinetic study using a non-human primate model showed that the serum half-life of SAFA-anti-CD40L is approximately 10 days, SAFA may have a serum half-life of approximately 2 weeks in humans, although this calculation is only an assumption (12, 27).

*In vivo*, we investigated the effect SAFA-FSH on spermatogenesis in a rat model of hypogonadism. Through several preliminary experiments, we successfully created a hypogonadism model in male SD rats, whose sperm counts did not respond to hCG alone (28). We confirmed that sperm production could be restored when SAFA-FSH was injected over a longer cycle than that when recombinant FSH was injected. The injection cycle of SAFA-FSH could be extended to once every 10 days, which is approximately four times longer than that of recombinant FSH treatment at intervals of thrice per week. The increase in testis weight and sperm count observed in this study suggests that SAFA-FSH could effectively replace recombinant FSH in the treatment regimen of adult men with HH desiring fertility (8). However, sperm production was unexpectedly restored after recombinant FSH treatment at intervals of once every 5 days. Although we showed that SAFA-FSH was effective when administered once every 10 days, comparative experiments with a wider injection cycle are needed in the future. The assays in this study were performed only on animals. Clinical trials are needed to determine whether these results can be extrapolated to humans. SAFA-FSH is a valuable alternative to recombinant FSH and may have great potential for therapeutic applications.

SAFA-FSH is intended to replicate the mechanism of the current thrice per week recombinant FSH treatments used to enhance spermatogenesis, but with a reduced number of administrations. It is clear from our study that this newly developed sustained FSH exerts pharmacological and physiological effects similar to those of recombinant FSH at the FSH receptor. SAFA-FSH can also be applied to women undergoing infertility treatment through comparative experimental analysis with recombinant FSH for ovarian weight gain and ovulation. The half-life of SAFA-FSH is expected to be sufficiently long to sustain the entire therapeutic period (29, 30).

This study had several limitations. First, the bioactivity of SAFA-FSH was lower than that of recombinant FSH, probably because of steric hindrance. Although recombinant FSH was used as a commercial material, SAFA-FSH was tested with an experimental material with a purity of > 95%. The manufacturing of high-purity materials and optimal formulation design for long-term storage are required. Second, we used genetically modified TM4-FSHR cells which may have altered their responsiveness to stimuli. Primary cultures of Sertoli cells provide an interesting model to study how signaling pathways induced (22, 31–33). However, primary cultured Sertoli cells frequently do not maintain their functions for prolonged periods of time in culture. The purpose of our *in vitro* study was to assess the bioactivity of SAFA-FSH versus recombinant FSH, and showed SAFA-FSH can activate cells similar to recombinant FSH. Third, we could not show that the half-life was very long, as expected from the pharmacokinetic study in rats. Considering the half-life of human serum albumin (3 weeks), the expected half-life of SAFA-FSH in humans is much longer because the half-life of SAFA-FSH in our study was slightly shorter than that of endogenous rat serum albumin (34). Fourth, safety tests were not conducted. SAFA is a substance of human origin and is expected to have no side effects, such as platelet activation, due to the absence of an Fc domain. However, further studies are required to confirm this. Fifth, unexpectedly, we observed that recombinant FSH, which was used as a reference, restored sperm count at intervals of once every 5 days and hence, should be compared at intervals of once every 10 days. Although further comparative studies are needed for confirmation, we showed that the injection cycle of SAFA-FSH can be extended to once every 10 days. Owing to its different kinetic profile, SAFA-FSH can replace multiple injections of FSH by promoting sustained sperm production.

In conclusion, SAFA-FSH can activate cells similar to recombinant FSH, and because it has a longer half-life, the drug concentration is maintained without frequent injections compared to recombinant FSH, thereby increasing the utility of long-acting hormone preparations. In this study, we demonstrated the successful development of SAFA-FSH using SAFA technology and believe that our approach can provide many benefits to patients in the future by generating highly effective and long-acting therapeutic biologics at reasonably affordable prices.

## Data availability statement

The original contributions presented in the study are included in the article/supplementary material. Further inquiries can be directed to the corresponding author.

## Ethics statement

The animal study was reviewed and approved by the Institutional Animal Care and Use Committee (IACUC, Yonsei University Health System, approval number 2021-0087).

## Author contributions

DK, CK, MS, SH-C, and EL contributed to the study conception and design. SL, YC, MK, HC, JA, KL, JH, and SC organized the database. SL performed the statistical analyses. DK wrote the first draft of the manuscript. DK and SL wrote sections of the manuscript. All authors contributed to manuscript revision and read and approved the submitted version.
